# Associations of behavioural risk factors and health status with changes in physical capability over 10 years of follow-up: the MRC National Survey of Health and Development

**DOI:** 10.1136/bmjopen-2015-009962

**Published:** 2016-04-18

**Authors:** Rachel Cooper, Graciela Muniz-Terrera, Diana Kuh

**Affiliations:** MRC Unit for Lifelong Health and Ageing at UCL, University College London, London, UK

**Keywords:** ageing, physical capability, age-related change, behavioural risk factors, health status

## Abstract

**Objectives:**

(1) To describe changes in objective measures of physical capability between ages 53 and 60–64 years; (2) to investigate the associations of behavioural risk factors (obesity, physical inactivity, smoking) and number of health conditions (range 0–4: hand osteoarthritis (OA); knee OA; severe respiratory symptoms; other disabling or life-threatening conditions (ie, cancer, cardiovascular disease, diabetes)) at age 53 years with these changes.

**Design:**

Nationally representative prospective birth cohort study.

**Setting:**

England, Scotland and Wales.

**Participants:**

Up to 2093 men and women from the Medical Research Council National Survey of Health and Development, who have been followed-up since birth in 1946, and underwent physical capability assessments performed by nurses following standard protocols in 1999 and 2006–2010.

**Main outcome measures:**

Grip strength and chair rise speed were assessed at ages 53 and 60–64 years. Four categories of change in grip strength and chair rise speed were identified: decline, stable high, stable low, a reference group who maintained physical capability within a ‘normal’ range.

**Results:**

Less healthy behavioural risk scores and an increase in the number of health conditions experienced were associated in a stepwise fashion with increased risk of decline in physical capability, and also of having low levels at baseline and remaining low. For example, the sex and mutually adjusted relative-risk ratios (95% CI) of being in the stable low versus reference category of chair rise speed were 1.58 (1.35–1.86) and 1.97 (1.57–2.47) per 1 unit change in behavioural risk score and health indicator count, respectively.

**Conclusions:**

These findings provide evidence of the associations of a range of modifiable factors with age-related changes in physical capability. They suggest the need to target multiple risk factors at least as early as mid-life when aiming to promote maintenance and prevent decline in physical capability in later life.

**Strengths and limitations of this study**

- This study characterises age-related changes in objective measures of physical capability between mid-life and early old age, across a life stage when there may be more opportunity to effectively intervene to prevent decline and promote maintenance.
- This is the first study to examine the mutually adjusted associations of behavioural risk factors and indicators of health status with changes in objective measures of physical capability.
- Findings suggest modifiable targets for intervention in mid-life, which focus on the prevention of decline and the promotion of maintenance in physical capability.
- The study sample was selected at birth to be nationally representative, and after 64 years it remained so in many respects. However, only those participants who were assessed at ages 53 and 60–64 years could be included in analyses, and it was necessary to exclude people who had been assessed at age 53 years but subsequently died.

## Introduction

Maintaining physical capability, the ability to perform the physical tasks of daily living, is an important component of healthy ageing.^1^ ^2^ However, age-related declines in physical capability are widely reported from mid-life onwards^3–5^ suggesting that opportunities for maintenance may be limited. An acknowledged limitation of cross-sectional data showing that older people have lower mean levels of physical capability than younger people on whom these reports are often based,^6–9^ is that they cannot distinguish age from cohort and period effects. Further, even when repeated data within individuals are available there is often a specific focus on modelling mean decline^10–16^ with methods employed that assume similar patterns of change for all. Thus interindividual differences in the patterns of within-individual change in physical capability observed over time may be overlooked. Where differences between individuals in the scale and direction of within-individual change in physical capability have been described,^3^ ^17^ ^18^ these suggest that a significant proportion of older people may not be experiencing meaningful decline in any specified observation period.

In light of evidence that patterns of within-individual change in physical capability may be heterogeneous, consistent with recent findings for other healthy ageing phenotypes,^19^ it seems plausible to distinguish four groups. Those experiencing decline in physical capability, those with relatively high levels at baseline which are maintained and those with relatively low levels at baseline who remain low may be compared and contrasted with those maintaining physical capability levels within a ‘normal’ range. Studying how these groups differ with respect to modifiable factors may present new opportunities for intervention focusing on the promotion of healthy ageing.

That a range of behavioural risk factors and indicators of health status are associated with physical capability and its age-related decline is well recognised.^13^ ^20–24^ However, a growing body of evidence highlights the benefits of considering the co-occurrence of these factors and examining their combined impact on different ageing phenotypes,^25^ including self-reported and performance-based measures of physical capability.^26–30^ However, few studies have assessed the mutually-adjusted associations of behavioural risk factors and health status together and none have related these to different patterns of change in objective measures of physical capability.

Using data from the Medical Research Council (MRC) National Survey of Health and Development (NSHD), a nationally representative British birth cohort study, the objectives of this paper were to: (1) describe the changes observed in objective measures of physical capability (grip strength and chair rise speed) from ages 53 to 60–64 years and the variability between individuals in these within-individual changes; (2) identify four prespecified categories of change in physical capability and; (3) investigate the associations of behavioural risk factors and indicators of health status with these categories of change.

## Materials and methods

The NSHD is a socially stratified sample of 5362 singleton births (2547 males and 2815 females) that took place in 1 week of March 1946 in mainland Britain, with regular follow-up across life.^31^ ^32^ During the two most recent waves of data collection, in 1999 (at 53 years) and 2006–2010 (at 60–64 years), physical capability was assessed using performance-based measures.

At age 53 years, 3035 participants were successfully contacted, of whom 2984 received a home visit from a trained nurse. At age 60–64 years, 2856 eligible participants (those known to be alive, living in England, Scotland or Wales, and who had not permanently refused to participate) were invited for assessment at one of six clinical research facilities (CRFs), or to be visited by a research nurse at home, of whom 2229 were assessed (1690 at a CRF and 539 at home). See online supplementary appendix figure 1 for reasons for losses to follow-up.

10.1136/bmjopen-2015-009962.supp1 Supplementary appendices

Relevant ethical approval was received; assessment in 1999 was approved by the North Thames Multi-Centre Research Ethics Committee, and in 2006–2010 by the Central Manchester Local Research Ethics Committee and the Scottish A Research Ethics Committee. All participants gave informed consent.

### Ascertainment of physical capability

Grip strength and chair rise speed were assessed at ages 53 and 60–64 years by trained nurses using standardised protocols as described elsewhere^33^ and summarised here.

At both ages, grip strength was measured isometrically using a Nottingham electronic handgrip dynamometer. The dynamometers were calibrated at the start of testing using a back-loading rig, and are accurate, linear and stable to ±0.5 kg.^34^ The intraparticipant retest variability for maximal voluntary tests of strength in those unused to such measurements is approximately ±9%.^35^ At age 53 years, four values (two in each hand), and at age 60–64 years, six values (three in each hand) were recorded. To ensure comparability between ages, the highest value achieved at age 53 years and the highest of the first four values recorded at age 60–64 years were used in analyses. At both ages, the time taken to rise from a sitting to a standing position with straight back and legs, and then sit down again 10 times as fast as possible was measured using a stopwatch. The times recorded were used to calculate chair rise speed (ie, number of rises (ie, 10)/time taken (in minutes)). For both tests, nurses recorded if a study participant was unable or unwilling to perform the test, and the reason for this (eg, health reasons, technical problems).

Four categories of change in grip strength and chair rise speed were identified as follows: (1) sex-specific SD scores (mean=0, SD=1) of grip strength and chair rise speed at each age (based on the distributions at that age) were derived in the samples with valid measures at both ages (n=1896 grip strength, n=1885 chair rise speed); (2) each SD score was categorised: <−1SD; −1 to 1 SD; >1SD; (3) the categorised SD scores for ages 53 and 60–64 years were cross-tabulated, and categories of change identified as shown in table 1. These categories were: (1) reference (ie, maintained physical capability within ‘normal’ range); (2) decline; (3) stable high; (4) stable low. Participants with valid values at age 53 years who were unable to complete the test for health reasons at age 60–64 years were included in the ‘decline’ category (n=41 grip strength, n=88 chair rise speed), and participants who were unable to complete the test for health reasons at both ages were included in the ‘stable low’ category (n=5 grip strength, n=38 chair rise speed).

**Table 1:** Four categories of change derived by cross-tabulating sex-specific SD scores for grip strength and chair rise speed at ages 53 and 60–64 years in the Medical Research Council National Survey of Health and Development

Grip strength/chair rise speed SD score at age	60–64 years		
53 years	<−1 SD	−1 SD to 1 SD	>1 SD
**<−1 SD**	**4.** Stable low	**1.** Reference	**1.** Reference
**−1 SD to 1 SD**	**2.** Decline	**1.** Reference	**1.** Reference
**>1 SD**	**2.** Decline	**2.** Decline	**3.** Stable high

Sex-specific SD scores calculated using the distributions of grip strength and chair rise speed at each age among the sample with valid measures at both ages.

### Explanatory factors

To create a cumulative behavioural risk factor score, three behavioural risk factors assessed at age 53 years that were associated with physical capability at this age were selected a priori: overweight and obesity; physical inactivity; smoking.^33^ ^36–38^ Height (cm) and weight (kg) were measured by nurses, and body mass index (BMI) (kg/m^2^) was calculated. For those people with a valid weight measure but missing height (n=5), height was imputed using a measure from age 43 years. BMI was categorised into three groups: normal weight (<25 kg/m^2^); overweight (25–29.99 kg/m^2^); obese (≥30 kg/m^2^). Participants were asked to report whether or not they had participated in any sports, vigorous leisure activities or exercises in their spare time, not including getting to and from work, in the past 4 weeks, and if so, on how many occasions they had done these activities. This was categorised into three groups: inactive (no participation); moderately active (participated 1–4 times); most active (participated ≥5 times). Smoking status was categorised as: current; ex; never smoker. Each of these factors was coded: 0=most healthy (ie, normal weight, most active, and never smoker, respectively); 1=intermediate (ie, overweight, moderately active, and ex-smoker, respectively) and; 2=least healthy (ie, obese, inactive, smoker, respectively). The three factors were then summed to create a total score ranging from 0 (most healthy) to 6 (least healthy).

#### Health status

Four indicators of health status at age 53 years that were cross-sectionally associated with physical capability^33^ were selected a priori: hand osteoarthritis (OA); knee OA; severe respiratory symptoms and other disabling or life-threatening conditions. During the home visit at 53 years, trained nurses conducted clinical examinations of the hands and knees using validated criteria. During assessment of the hands, the presence of Heberden nodes, Bouchard nodes and squaring at the carpometacarpal joint were identified, and hand OA was defined as involvement of at least one joint.^39^ Knee OA was defined using the American College of Rheumatology criteria for the clinical diagnosis of idiopathic knee OA,^40^ namely knee pain in either knee on most days for at least 1 month in the last year, and at least two of the following: stiffness, crepitus, bony tenderness and bony enlargement.^41^ Respiratory symptoms were assessed using the UK Medical Research Council's standardised questions.^42^ A group with the most severe symptoms was identified who reported one or more of the following: a wheezy or whistling chest most days or nights; usually bringing up phlegm or coughing in the morning or during the day or night in winter for at least 3 months each year; or more than one chest illness in the past 3 years that kept them off work or indoors for 1 week or more. Those participants who at 53 years reported cancer in the previous 10 years, diabetes at any time up to and including age 53 years, or cardiovascular disease (defined as having: a heart attack or stroke ever, aortic stenosis or valvular disease in the past 10 years, doctor-diagnosed angina or Rose angina grade I or II, or intermittent claudication), were categorised as having other potentially disabling or life-threatening conditions. To assess the cumulative influence of comorbidities, a variable was derived that indicated the number of health status indicators each participant reported ranging from 0 (no reported health problems) to 4 (all 4 indicators reported).

### Statistical analyses

Descriptive analyses were used to explore the mean levels of grip strength and chair rise speed at ages 53 and 60–64 years in the total sample, and in each of the four categories of change. Latent change score models^43^ ^44^ using the maximum data available provided an estimate of the between-individual variability in the level of within-individual change observed.

Using multinomial logistic regression models, the associations of the behavioural risk factor score and the health indicator count with the different categories of change (vs the reference group) in grip strength and chair rise speed were tested. Relative-risk ratios (RRR) were estimated for unit changes in the behavioural risk factor score and health indicator count with deviations from linearity formally tested to ensure that assumptions of linearity were met. Initial models were adjusted for sex, with sex interactions also formally tested. A subsequent model included mutual adjustment for behavioural risk and health status. Models were run on the samples with complete data on change and both sets of risk factors (N=1906, grip strength and N=1975, chair rise speed).

Sensitivity analyses were performed to test the sex-adjusted associations of each component of the behavioural risk factor score and the health indicator count with changes in grip strength and chair rise speed.

## Results

Mean levels of grip strength and chair rise speed were lower at age 60–64 years than at 53 years in men and women (table 2). Latent change score models estimated mean changes in: grip strength (kg) of −3.23 (95% CI −3.88 to −2.59) for men and −2.07 (−2.51 to −1.63) for women; and chair rise speed (stands/minute) of −5.91 (−6.40 to −5.42) for men and −5.23 (−5.71 to −4.74) for women. Estimates of variance and covariance in these models were statistically significant (p<0.001) (see online supplementary appendix 2) showing a high degree of between-individual variability in within-individual change. When changes in grip strength and chair rise speed were categorised into four groups, approximately 20% of participants were classified as showing clear evidence of decline, approximately 5% were classified as stable high, and a similar proportion as stable low (table 3).

**Table 2:** Characteristics of the Medical Research Council National Survey of Health and Development stratified by sex (sample restricted to those with data on change in grip strength and/or chair rise speed (maximum N=2093)).

	Men		Women		
	N*	Mean (SD) or %	N*	Mean (SD) or %	p Value†
*Physical capability at ages 53 and 60–64 years*					
Grip strength at 53 years (kg)	958	47.8 (12.1)	1067	28.1 (8.0)	<0.001
Grip strength at 60–64 years (kg)	934	44.6 (11.6)	1020	26.0 (7.4)	<0.001
Chair rise speed at 53 years (stands/min)	939	32.1 (10.2)	1049	30.8 (9.3)	0.003
Chair rise speed at 60–64 years (stands/min)	919	26.4 (7.2)	1029	25.5 (7.9)	0.01
*Behavioural risk factors and indicators of health status at age 53 years*					
Body mass index (BMI)					
Obese	983	20.2	1101	23.4	<0.001
Physical activity					
Inactive	985	42.8	1108	45.4	0.22
Smoking status					
Current smoker	985	18.4	1108	19.3	<0.01
Behavioural risk factor score (range 0–6)	983	2.9 (1.3)	1101	2.8 (1.5)	0.06
Hand osteoarthritis	985	18.9	1108	30.1	<0.001
Knee osteoarthritis	975	7.1	1092	12.2	<0.001
Severe respiratory symptoms	985	16.4	1107	16.9	0.74
Other disabling/life-threatening conditions‡	984	8.5	1107	9.4	0.49
Health indicator count (range 0–4)	974	0.5 (0.7)	1091	0.7 (0.8)	<0.001
≥1 health indicator reported		40.6		50.4	

For brevity descriptive statistics for only the ‘highest risk’ category of each categorical variable are presented. Categorisations used are as follows: BMI (normal weight (<25 kg/m^2^); overweight (25–29.99 kg/m^2^); obese (≥30 kg/m^2^)); physical activity (inactive (no reported leisure time physical activity); moderate (1–4 times per week); high (5 or more times per week)); smoking status (current, ex, never smoker).

All health status indicators were coded as binary variables, and percentages shown are the proportion of people in each category with the specified condition/s.

*N varies due to missing data.

†p Values from formal tests of sex difference.

‡Includes cancer, diabetes and cardiovascular disease. Of those with at least one condition, 94% of men and 98% of women had only 1 of the 3 conditions (no men and only 1 woman reported all 3 conditions).

**Table 3:** Mean levels of grip strength and chair rise speed at ages 53 and 60–64 years in each category of change in the Medical Research Council National Survey of Health and Development

	Men			Women		
	N* (%)	Mean (SD)		N* (%)	Mean (SD)	
		at 53 years	at 60–64 years		at 53 years	at 60–64 years
Grip strength (kg)						
Reference	649 (70.5)	46.1 (9.6)	46.6 (8.5)	688 (67.3)	26.5 (5.9)	27.3 (5.8)
Decline	169 (18.4)	53.6 (12.2)	35.9 (11.4)	231 (22.6)	31.6 (8.3)	20.5 (6.5)
Stable high	53 (5.8)	68.1 (6.5)	64.7 (7.2)	54 (5.3)	41.9 (8.4)	38.8 (4.1)
Stable low	49 (5.3)	28.6 (6.3)	27.2 (4.3)	49 (4.8)	16.8 (2.5)	15.5 (2.6)
Chair rise speed (stands/min)						
Reference	648 (68.4)	29.8 (6.2)	26.9 (5.3)	771 (72.5)	29.2 (6.0)	26.2 (6.8)
Decline	195 (20.6)	36.6 (13.5)	21.6 (5.9)	202 (19.0)	35.8 (12.8)	21.3 (6.9)
Stable high	56 (5.9)	51.1 (7.9)	42.0 (6.2)	37 (3.5)	50.4 (7.8)	42.0 (8.4)
Stable low	49 (5.2)	18.7 (2.3)	16.7 (2.0)	53 (5.0)	17.8 (2.9)	15.1 (2.5)

See table 1 for details of how categories are defined.

In total sample with valid measures at both ages, mean (SD) sex-specific values are as follows:

Grip strength (kg): men (N=908): 53 years=47.9 (12.1); 60–64 years=44.7 (11.6); women (N=988): 53 years=28.2 (7.9); 60–64 years=26.0 (7.4).

Chair rise speed (stands/min): men (N=893): 53 years=32.2 (10.2); 60–64 years=26.5 (7.2); women (N=992): 53 years=31.2 (9.2); 60–64 years=25.7 (7.9).

*Total N in each category. Ns for presented means may vary due to inclusion in these categories of: (1) participants with valid values at 53 who were unable to complete the test for health reasons at 60–64 years in the ‘evidence of decline’ category (n=41 for grip strength (12 men, 29 women), n=88 for chair rise speed (37 men, 51 women)) and; (2) participants who were unable to complete the test for health reasons at both ages in the ‘stable low’ category (n=5 for grip strength (0 men, 5 women), n=38 for chair rise speed (18 men, 20 women)).

Increases in the behavioural risk factor score and health indicator count were both associated with increased risk of decline in grip strength when compared with the reference category (table 4). These associations were maintained after mutual adjustment. Associations were also found between the behavioural risk factor score, health indicator count and risk of being in the stable low category. There was some evidence to suggest that poorer health status was more strongly associated with risk of being in the stable low category (RRR=1.36 (95% CI 1.06 to 1.75)) than of being in the decline category (RRR=1.15 (1.00 to 1.33)).

**Table 4:** Sex-adjusted associations of behavioural risk factor score and health indicator count at age 53 years with categories of change in grip strength and chair rise speed in the Medical Research Council National Survey of Health and Development (sample restricted to those with complete data on change and both composite scores)

	Relative-risk ratios (95% CI) of being in specified category of change relative to reference category					
	Grip strength (N=1906)			Chair rise speed (N=1975)		
	Stable high	Stable low	Decline	Stable high	Stable low	Decline
Behavioural risk factor score* (per 1 unit increase)						
Model 1	0.89 (0.77 to 1.02)	1.19 (1.03 to 1.38)	1.14 (1.05 to 1.23)	0.69 (0.58 to 0.81)	1.73 (1.48 to 2.03)	1.15 (1.06 to 1.24)
Model 2	0.90 (0.78 to 1.04)	1.14 (0.99 to 1.33)	1.12 (1.04 to 1.22)	0.69 (0.59 to 0.81)	1.58 (1.35 to 1.86)	1.12 (1.03 to 1.21)
Health indicator count† (per 1 unit increase)						
Model 1	0.77 (0.57 to 1.03)	1.43 (1.13 to 1.83)	1.20 (1.04 to 1.39)	0.81 (0.58 to 1.13)	2.30 (1.84 to 2.86)	1.34 (1.16 to 1.55)
Model 2	0.79 (0.58 to 1.07)	1.36 (1.06 to 1.75)	1.15 (1.00 to 1.33)	0.90 (0.64 to 1.27)	1.97 (1.57 to 2.47)	1.29 (1.12 to 1.50)

Maximum number of participants in each category of change shown in table 3 however, 36 participants shown in table 3 had to be excluded from analyses presented in table 4 due to missing data on behavioural risk factors and/or health indicators.

Model 1: adjusted for sex (no evidence of sex interactions (p≥0.34) or deviations from linearity (p≥0.06) when formally tested).

Model 2: adjusted for sex and health indicator count or behavioural risk factor score (as appropriate).

*Behavioural risk factor score (range 0 (most healthy (ie, normal weight, most active, never smoker)) to 6 (least healthy (ie, obese, inactive, current smoker))).

†Health indicator count (range 0 (no reported health problems) to 4 (all 4 health indicators reported (ie, hand osteoarthritis (OA), knee OA, severe respiratory symptoms, and other disabling/life-threatening conditions)).

For chair rise speed, increases in the behavioural risk factor score and health indicator count were both associated with increased risks of being in the decline or stable low category, and with reduced risk of being in the stable high category when compared with the reference category. These associations were attenuated but maintained after mutual adjustment. Associations with risk of being in the stable high category appeared to be stronger for behavioural risk factors than for health status. On the basis of a qualitative assessment of the size of the effect estimates (table 4), there was also some evidence to suggest that the behavioural risk factor score and health indicator count were more strongly associated with risk of being in the stable low category, than with risk of being in the decline category.

In sensitivity analyses, similar patterns of association were found for the majority of individual components of the behavioural risk factor score and health indicator count (see online supplementary appendix 3), although some associations were weaker.

## Discussion

In a large nationally representative study, declines in mean levels of grip strength and chair rise speed between ages 53 and 60–64 years were observed. The changes in these summary statistics with age, disguised a large amount of variability between individuals in the levels of within-individual change experienced. In addition to those who had clearly declined, three other groups that appeared to have maintained their capability over time but had different baseline levels of capability and, hence, different health prospects, could be identified. There was evidence that people in each of these categories had different risk factor profiles. Less healthy behavioural risk scores and an increase in the number of health conditions experienced at baseline were both associated with increased risk of decline and of having low levels of capability at baseline and remaining low when compared with the reference group of participants who maintained physical capability within a ‘normal’ range. Those who had high levels of physical capability at baseline and retained these were more likely to have healthier behavioural risk scores and fewer health conditions than those in the reference group.

### Comparison with other studies

The mean annual changes in grip strength observed in the NSHD were similar to those reported in a Finnish study at a similar age.^14^ Observed changes in mean levels of chair rise speed were slightly greater than expected, based on cross-sectional estimates.^6^ ^7^ However, it has been shown that cross-sectional data may underestimate age-related change.^11^ ^18^ That we found that the summary estimates mask significant between-individual variability in the levels of within-individual change experienced confirms findings from other studies that have described this.^3^ ^17^ ^18^

These findings build on, and extend those from previous studies, that have explored the associations of behavioural risk factors and/or health status with physical capability.^26–30^ Like these studies, we also found evidence of dose–response effects when combining different behavioural risk factors in the same score, and when investigating number of comorbidities reported. However, this is the first study to examine the associations of both sets of factors with different patterns of change in objective measures of physical capability in mutually adjusted models.

### Explanation of findings

Underlying explanations of the associations between each individual component of these scores and physical capability have been described previously.^33^ ^36–38^ Consistent with findings from other studies,^25^ clearer evidence of associations was found when using these composite scores than when examining each component separately, which is likely to be explained by the co-occurrence of these factors.

Behavioural risk factors and indicators of health status were associated not only with risk of decline but also with risk of being in the stable low and stable high categories. This is likely to be partially explained by the influence of behavioural risk factors and underlying disease processes on the development and maintenance of physical capability in early to mid-life as well as on age-related declines. That the health indicator count was more strongly associated with risk of being in the stable low category than in the decline category suggests that having low levels of physical capability in mid-life may be a manifestation of underlying disease processes that started earlier in life and may have influenced development. It also indicates that the negative impact on physical capability of conditions such as OA, usually reported in older populations,^45^ may already be manifest by mid-life.

### Methodological considerations

A key strength of our analyses was the availability of data on within-individual change in two widely used objective measures of physical capability. As data were collected in mid-life and early old age, this allowed us to study changes across a life stage when there may be more opportunity to effectively intervene to prevent decline and promote maintenance. By studying change across this specific age period, we could also attempt to distinguish between two groups with low levels of physical capability in old age who will have followed different life course trajectories; those who had achieved relatively high levels of capability earlier in life but then experienced decline in early old age, and those who have experienced poor development (and/or an earlier onset of decline), and so have had relatively low levels of capability across a longer period (and so had less ‘opportunity’ to decline during the study observation period).

The method of categorising change in physical capability was selected a priori to capture these and other expected differences that may be meaningful in the context of healthy ageing. This approach was also used with the aim of avoiding or minimising the impact of some of the limitations inherent in modelling change continuously when data are only available at two time points,^46^ ^47^ including regression to the mean, measurement error, practice effects and inability to identify the shape of change. While established methods of categorising change scores were considered,^48^ the strict criteria applied when using these other methods resulted in only those people with very high levels of absolute decline who were more likely to have had high levels of capability at baseline, being distinguished from everyone else. Further, these other methods resulted in the creation of a heterogeneous ‘no decline’ category which grouped together those with a wide range of different baseline levels of capability, including those with the highest and lowest levels, who have very different health prospects. The strengths of our approach are likely to outweigh limitations including potential misclassification and reduced statistical power (particularly to detect associations in the relatively small stable high and low groups). That the main findings were largely unchanged when models were rerun using an alternative method to identify the four categories of change (details available on request), supports this and suggests that our findings are robust.

The NSHD study sample was selected at birth to be nationally representative, and after 64 years, it remained so in many respects.^49^ However, the fact that only those participants who were assessed at ages 53 and 60–64 years could be included in our analyses needs to be taken into account when interpreting findings. It is also necessary to consider that bias could have been introduced by the exclusion of people who had been assessed at age 53 years but subsequently died. Previous analyses in the NSHD have shown that those who had weaker grip strength and slower chair rise speed at 53 years had higher rates of all-cause mortality over 13 years of follow-up.^50^ However, a Finnish study applied methods to take account of the effect of this right censoring due to death when estimating annual changes in grip strength across adulthood. This study found little impact of applying this method on the estimates produced before age 65 years,^14^ suggesting that exclusion of those participants who died between ages 53 and 60–64 years in our analyses is unlikely to have introduced significant bias.

In these analyses, we chose a priori to focus on three key behavioural risk factors (ie, physical inactivity, obesity and smoking). However, it is acknowledged that other behavioural risk factors including high alcohol intake and poor diet quality may also be important, and warrant investigation in future analyses.

### Implications

Our finding of associations of a behavioural risk factor score and health indicator count with changes in physical capability provides evidence of the importance of the effects of both these sets of modifiable factors. It suggests the need to take a holistic approach to intervention and to target multiple risk factors when aiming to promote maintenance and prevent decline in physical capability. That these factors were associated not only with risk of decline but also with risk of being in the stable low category highlights the need to monitor physical capability in mid-life to identify opportunities for early intervention; opportunities may already have been missed if no action is taken until later in life.

## Conclusions

This study has demonstrated that change in physical capability between mid-life and early old-age, is a heterogeneous experience. It suggests that age-related decline may not be entirely inevitable, and is potentially modifiable. That there was consistent evidence of associations between a behavioural risk factor score (incorporating obesity, smoking and inactivity) and a health indicator count (incorporating a range of potentially disabling health conditions) at mid-life with different patterns of change in physical capability suggests potential opportunities for intervention to prevent decline and promote maintenance of physical capability.
