# Correction: *Associations of behavioural risk factors and health status with changes in physical capability over 10 years of follow-up: the MRC National Survey of Health and Development*

**DOI:** 10.1136/bmjopen-2015-009962corr1

**Published:** 2018-05-05

Cooper R, Muniz-Terrera G, Kuh D. Associations of behavioural risk factors and health status with changes in physical capability over 10 years of follow-up: the MRC National Survey of Health and Development. *BMJ Open* 2016;6:e009962. doi: 10.1136/bmjopen-2015-009962

The first line of the methods reads:

‘The NSHD is a socially stratified sample of 5362 singleton births (2547 males and 2815 females)’

however, this should have read:

‘The NSHD is a socially stratified sample of 5362 singleton births (2815 males and 2547 females)’.

