# Supplementary material for: Associations of behavioural risk factors and health status with changes in physical capability over 10 years of follow-up: the MRC National Survey of Health and Development
Source: BMJ Open. 2016 Apr 18;6(4):e009962. doi: 10.1136/bmjopen-2015-009962 (PMC4838696; doi:10.1136/bmjopen-2015-009962)
Supplement: Supplementary appendices [file bmjopen-2015-009962supp.pdf]

Appendix 1: Flow diagram of participation in clinical assessments at ages 53 and 60-64 years in the MRC National Survey of Health and Development

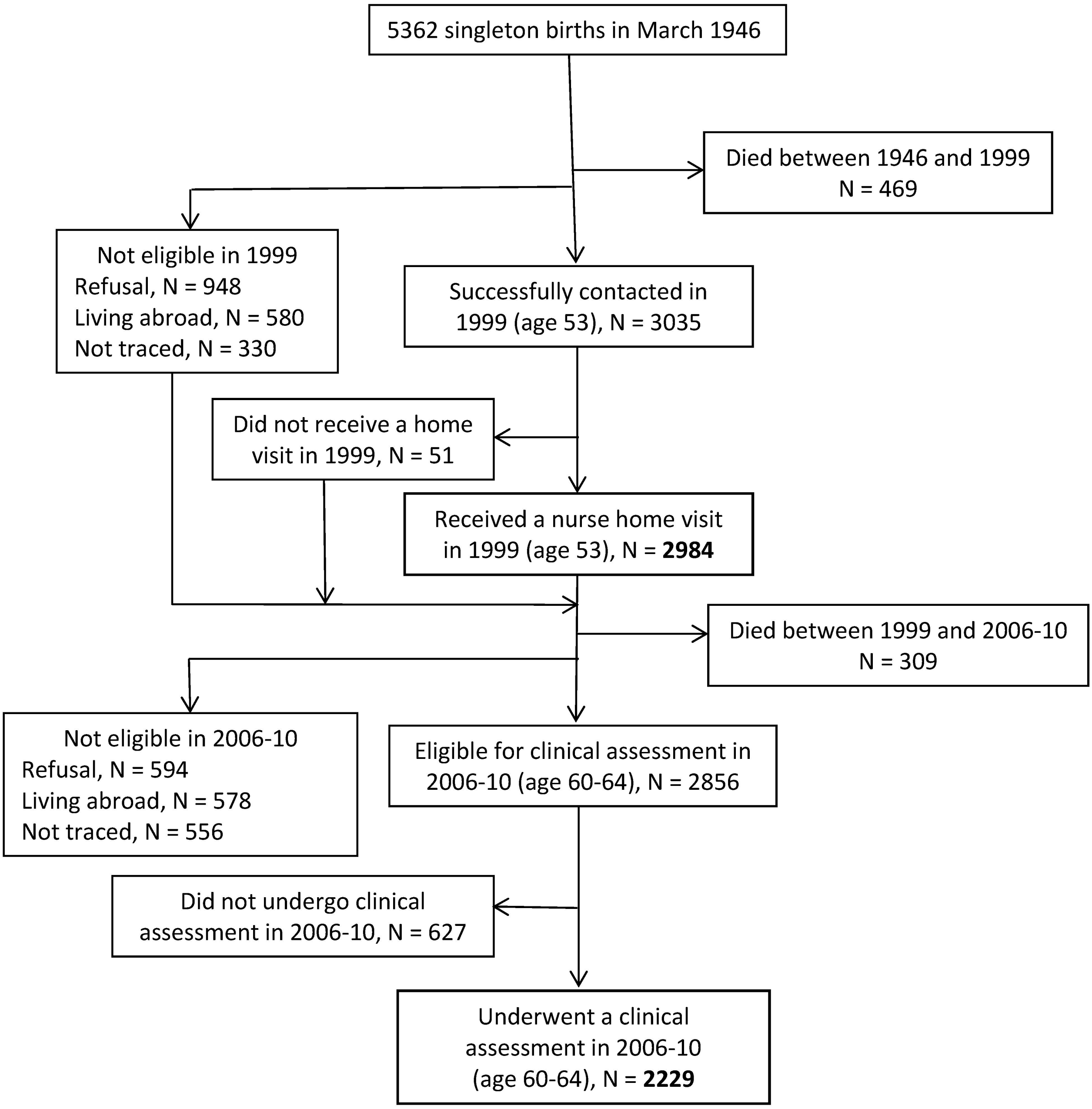

Appendix 2: Estimates from latent change score models describing the estimated mean changes in grip strength and chair rise speed in men and women between ages 53 and 60-64 years in the MRC National Survey of Health and Development

(T_1 = 53 years; Delta = change between 53 and 60-64 years; $T_2 = 1.T_1$ + Delta + error)

		Model estimated means		
	**N**	**T_1**	**Delta**	**Covariance**
**Grip strength (kg)**				
Men	1383	47.90 (47.35, 48.45)	-3.23 (-3.88, -2.59)	-85.20 (-94.02, -76.38)
Women	1406	28.00 (27.65, 28.36)	-2.07 (-2.51, -1.63)	-43.46 (-47.43, -39.49)
**Chair rise speed (stands/min)**				
Men	1315	32.46 (31.97, 32.94)	-5.91 (-6.40, -5.42)	-74.85 (-81.10, -68.60)
Women	1355	30.68 (30.26, 31.10)	-5.23 (-5.71, -4.74)	-58.53 (-63.73, -53.32)

Using all available data from T_1 and T_2 for participants with:
1) valid data at T_1 AND valid data at T_2 (1896 Grip strength, 1885 Chair rise speed)
2) valid data at T_1 AND missing data at T_2 despite participation in data collection (due to being unable to perform the test for reasons other than health or having missing data) (102 Grip strength, 26 Chair rise speed)
3) valid data at T_1 AND missing data at T_2 due to non-response (not due to death) (648 Grip strength, 614 Chair rise speed)
4) valid data at T_2 and missing data at T_1 due to non-response or problems (other than health reasons) with measures (143 Grip strength, 145 Chair rise speed)

Appendix 3: Sex-adjusted[a] associations of individual behavioural risk factors and indicators of health status at age 53y with categories of change in grip strength and chair rise speed in the MRC National Survey of Health and Development

		Relative-risk ratios (95% CI) of being in specified category of change relative to reference category					
		Grip strength (N=1906)			Chair rise speed (N=1975)		
**Behavioural risk factors**		**Stable high**	**Stable low**	**Decline**	**Stable high**	**Stable low**	**Decline**
**BMI (kg/m^2)**	< 25	1.00	1.00	1.00	1.00	1.00	1.00
	25-29.99	1.27 (0.80, 2.00)	0.86 (0.53, 1.40)	1.18 (0.91, 1.53)	0.81 (0.51, 1.30)	1.54 (0.90, 2.65)	1.02 (0.79, 1.33)
	≥ 30	1.08 (0.62, 1.89)	1.19 (0.69, 2.03)	1.12 (0.82, 1.52)	0.69 (0.36, 1.30)	3.09 (1.77, 5.40)	1.40 (1.04, 1.89)
**Physical activity**	Most active	1.00	1.00	1.00	1.00	1.00	1.00
	Moderately active	0.91 (0.55, 1.50)	1.09 (0.54, 2.18)	1.19 (0.86, 1.65)	0.78 (0.46, 1.31)	1.38 (0.65, 2.97)	1.19 (0.87, 1.62)
	Inactive	0.51 (0.32, 0.82)	2.44 (1.48, 4.00)	1.53 (1.18, 1.97)	0.29 (0.17, 0.51)	3.84 (2.20, 6.70)	1.20 (0.93, 1.55)
**Smoking**	Never	1.00	1.00	1.00	1.00	1.00	1.00
	Ex	1.24 (0.79, 1.94)	0.87 (0.55, 1.39)	0.98 (0.75, 1.28)	0.79 (0.50, 1.25)	1.65 (0.96, 2.85)	0.99 (0.76, 1.29)
	Current	0.79 (0.41, 1.51)	0.84 (0.45, 1.56)	1.36 (0.99, 1.87)	0.35 (0.15, 0.81)	2.71 (1.48, 4.95)	1.66 (1.22, 2.27)
**Indicators of health status**							
**Hand OA**	No	1.00	1.00	1.00	1.00	1.00	1.00
	Yes	0.89 (0.56, 1.44)	1.23 (0.77, 1.95)	1.09 (0.84, 1.41)	0.83 (0.48, 1.44)	2.34 (1.53, 3.58)	1.20 (0.93, 1.56)
**Knee OA**	No	1.00	1.00	1.00	1.00	1.00	1.00
	Yes	0.51 (0.20, 1.27)	2.10 (1.18, 3.72)	1.19 (0.82, 1.73)	1.18 (0.53, 2.64)	4.07 (2.44, 6.80)	2.12 (1.49, 3.02)
**Severe respiratory symptoms**							
	No	1.00	1.00	1.00	1.00	1.00	1.00
	Yes	0.67 (0.36, 1.25)	1.32 (0.78, 2.22)	1.42 (1.07, 1.89)	0.48 (0.22, 1.05)	2.40 (1.52, 3.79)	1.58 (1.19, 2.09)
**Other disabling/life threatening health conditions**	No	1.00	1.00	1.00	1.00	1.00	1.00
	Yes	0.69 (0.30, 1.61)	2.15 (1.20, 3.87)	1.42 (0.97, 2.06)	0.96 (0.43, 2.12)	2.89 (1.70, 4.89)	1.00 (0.66, 1.51)

a. Formal tests of sex interaction, $p \geq 0.09$ with the exception of the associations between physical activity and changes in grip strength (p=0.04) and knee OA and changes in chair rise speed (p=0.05), in both cases associations were in the same direction in both sexes but effect sizes for PA and grip strength were of a higher magnitude among men than women and for knee OA and chair rise speed were of a higher magnitude among women than men (results available on request).
